# Trends in method-specific suicide in Brazil from 2000 to 2017

**DOI:** 10.1007/s00127-021-02060-6

**Published:** 2021-03-29

**Authors:** Keltie McDonald, Daiane Borges Machado, Luís F. S. Castro-de-Araujo, Lígia Kiss, Alexis Palfreyman, Maurício L. Barreto, Delanjathan Devakumar, Glyn Lewis

**Affiliations:** 1 Division of Psychiatry, University College London, 6th Floor Maple House, 149 Tottenham Court Road, London, W1T 7NF UK; 2 Center of Data and Knowledge Integration for Health (CIDACS), Salvador, Brazil; 3 Department of Global Health and Social Medicine, Harvard Medical School, Boston, MA USA; 4 The University of Melbourne, Department of Psychiatry, Austin Health, Heidelberg, VIC Australia; 5 Institute for Global Health, University College London, London, UK; 6 Institute of Collective Health, Federal University of Bahia, Salvador, Brazil

**Keywords:** Suicide, Mental health, Epidemiology, Ecological study, Public health

## Abstract

**Purpose:**

Understanding long-term patterns of suicide methods can inform public health policy and prevention strategies. In Brazil, firearm-related policies may be one salient target for suicide prevention. This study describes trends in method-specific suicide at the national and state-levels in Brazil, with a particular focus on firearm-related suicides.

**Methods:**

Brazilian mortality data for suicide and undetermined intent among people aged 10 years and older between 2000 and 2017 were obtained from the National Mortality Information System. We examined national and state-level trends in age-standardised suicide rates for hanging, self-poisoning, firearms, jumping from a high place, other, and unspecified methods. We also compared total rates of mortality from suicide and undetermined intent over the period. Applying Joinpoint regression, we tested changes in trends of firearm-specific suicide rates.

**Results:**

The total suicide rate increased between 2000 and 2017. Rates of hanging, self-poisoning by drugs or alcohol and jumping from a high place showed the largest increases, while firearm-specific suicide rates decreased over the study period. Trends in methods of suicide varied by sex and state.

**Conclusion:**

It is of public health concern that suicide rates in Brazil have risen this millennium. Restricting access to firearms might be an effective approach for reducing firearm-specific suicides, especially in states where firearm availability remains particularly high. Treatment and management of substance misuse may also be an important target for suicide prevention policies. More work is needed to understand the causes of rising suicide rates in Brazil and to improve the mental health of the population.

**Supplementary Information:**

The online version contains supplementary material available at 10.1007/s00127-021-02060-6.

## Introduction

Suicide is a leading cause of premature mortality and potential years of life lost worldwide. Approximately 800,000 people die by suicide each year, and this number is likely to be underreported [1]. The majority of global suicides occur in low and middle income countries [1]. In Brazil, the suicide rate is reported to have increased over the last two decades [2, 3], although, there is considerable variation in suicide rates within Brazil, with particularly high rates in men, indigenous people, and people aged 60 years and older [2].

Whilst trends in total suicides in Brazil have been studied (e.g. [2, 3, 15, 16]), patterns in the methods of suicide have received comparatively little attention. A recent ecological study examined trends in methods of suicide in 10–19-year-olds in Brazil between 2006 and 2015. Hanging (combined with drowning) was the most frequent method of suicide, followed by self-poisoning and use of firearms. Furthermore, deaths from hanging increased by approximately 15% over the study period, whilst self-poisoning and firearms decreased [4]. It remains unclear whether these trends in methods of suicide are generalisable to other age groups. More evidence is needed for the methods of suicide in the adult population.

Understanding current and emerging methods of suicide can inform effective prevention strategies. One of the most effective population-level strategies for suicide prevention is through limiting access to methods of suicide. In addition to access to means of suicide, the choice of method also depends on social and cultural factors [5]. Therefore, effective prevention through limiting access requires a good understanding of methods of suicide used in different regions and populations and their changes over time.

The total rate of death by firearms in Brazil is among the highest in the world, accounting for 21.5 deaths per 100,000 Brazilians in 2017 [6]. Ready access to firearms, whether legal or illegal, arguably facilitates deaths by firearms, including suicide. The lethality of firearms in suicide attempts is much higher than that of other methods such as bladed weapons or poison. Considering that most survivors of suicide attempts do not attempt suicide a second time, prevention of firearm-specific suicide can potentially reduce overall suicide mortality [17]. A national Disarmament Statute (Estatuto do Desarmamento; no. 10826) was passed on 22 December 2003, which introduced stricter criteria for the control of firearms. The federal law criminalised handling, trading or possession of materials for the production of weapons without a license. Some evidence suggests that the implementation of the statute coincided with a reduction in the number of hospitalisations and deaths due to firearms, including self-inflicted injuries, and homicides [7, 8].

This study aimed to investigate trends in method-specific suicide between 2000 and 2017 at the national and state-levels in Brazil, and to examine trends in firearm-specific suicide rates in relation to the introduction of a federal firearm policy.

## Methods

### Data

Mortality data were obtained from the National Mortality Information System (SIM), a database managed by the Ministry of Health in Brazil with information regarding all deaths in the country classified according to the International Classification of Diseases, 10th Edition (ICD-10) [9]. We extracted data on all deaths from external causes classified as intentional self-harm (X60–X84) and undetermined intent (Y10–Y34) from 2000 to 2017. The classification of suicide requires knowledge of the intent of the act; when suicide intent cannot be clearly established, an alternative verdict, often undetermined intent or accidental death, may be returned. For this reason, it is recognised that suicide rates are often underestimated in studies of suicide verdicts alone [10–12]. Although suicide research in Brazil usually includes only deaths from intentional self-harm, it is common in other countries to combine suicide with undetermined intent to account for potential misclassification of cause of death. Therefore, we also examined the sum of deaths from suicide and undetermined intent as a secondary outcome.

We excluded deaths amongst people under 10 years due to the very low rate of suicide in this young age group. Methods of suicide were categorised according to ICD-10 as: self-poisoning from drugs or alcohol (X60–X65), self-poisoning from pesticides (X68), self-poisoning from other poisons (X66, X67, X69), hanging or asphyxiation (X70), firearms (X72–X74), jumping from a high place (X80), other (X71, X75–X79, X81–X83), and unspecified methods (X84). Likewise, deaths from undetermined intent were classified into self-poisoning from drugs or alcohol (Y10–Y15), self-poisoning from pesticides (Y18), self-poisoning from other poisons (Y16–Y17, Y19), hanging or asphyxiation (Y20), firearms (Y22–Y24), jumping from a high place (Y30), other (X21, Y25–Y29, Y31–Y33), and unspecified methods (Y34).

Population estimates were obtained from the Brazilian Institute of Geography and Statistics (IBGE). All data are freely available online [13]. Population estimates for the corresponding age, sex, and municipality strata were only available for 2000 to 2012. Since the census in Brazil is only conducted every 10 years (most recently 2000 and 2010), population estimates for the remaining years were calculated by IBGE using interpolations and extrapolations until 2012. To estimate the population after 2012, following the IBGE’s strategy, we linearly extrapolated the population estimates in each stratum for each year from 2013 to 2017. The population data were appended to the mortality data, stratified by age group (10–14, 5-year age bands, 80 and older), sex, and municipality. For the analyses, data were aggregated to the national and state (*n* = 27) levels.

#### Missing data

The extracted data contained information from 168,703 deaths recorded as suicide among people aged 10 years and older from 2000 to 2017. We excluded from our analyses 25 (0.01%) suicides with missing sex information and 1246 (0.7%) with missing age information, yielding a total of 167,448 suicide deaths for analysis. For the same period, there were 189,179 deaths from external causes of undetermined intent among people 10 years and older. Of these, we excluded 480 (0.1%) deaths with missing sex information and 11,332 (6.0%) missing age information, yielding 177,822 deaths from undetermined intent for analysis (for additional details see Online Resource 1; Table S1).

### Statistical analysis

#### Summary of total and method-specific suicide rates

We summarised the numbers, percentages and rates per 100,000 inhabitants of total and method-specific suicide deaths by sex for the study period, as well as the percentage change between 2000 and 2017. To summarise temporal trends in mortality from suicide, we calculated age-standardised suicide rates per 100,000 for each method and year from 2000 to 2017. We also calculated age-standardised suicide rates by sex, state, and within larger age groups (10–19, 20–39, 40–59, and 60 years and older). To account for potential misclassification of suicidal intent, we produced total, sex-specific and method-specific rates per 100,000 for combined suicide and undetermined intent at the national-level. At the state-level, we visualised age- and sex-standardised suicide rates per 100,000 for 2000 and 2017, and the absolute change in rates from 2000 to 2017 (rate differences). Standardised rates were calculated using the World Health Organization (WHO) world standard population [14].

#### Trends in firearm-specific suicide rates

Joinpoint regression was used to estimate trends in age-standardised suicide rates by firearm for males and females. Joinpoint regression can be used to identify possible time points at which a trend significantly changes [15]. Permutation tests are performed to determine the minimum number of changes in trends (joinpoints) to best fit the data. Errors were calculated using weighted least squares to account for heteroscedasticity. We used a significance level of 0.05 for the permutation test with 4499 randomly permuted datasets (the minimum number of permutations suggested for greater consistency of *p* values). We used an adjusted permutation test, which is preferred in the presence of positive autocorrelated errors (the unadjusted test is favourable in the presence of large negative autocorrelated errors, which we did not suspect in our data) or when a more conservative test with minimal assumptions is desired. Using a piecewise log-linear model, estimates of annual percentage change (APC) with 95% intervals for trends were produced for each time period between joinpoints.

#### Software

Data management and statistical analyses were performed in Stata version 15 [16], and figures were produced in R [17]. Joinpoint regression was performed in Joinpoint Regression Program, version 4.8.0.1 [18].

## Results

### Total suicide rates

Among people ages 10 years and older in Brazil, the total age-standardised rate of combined suicide and undetermined intent was 10.82 (95% CI 10.80–10.86) per 100,000 from 2000 to 2017. The age-standardised rates of suicide and undetermined intent, separately, were 5.57 (95% CI 5.55–5.59) and 5.26 (95% CI 5.23–5.28) per 100,000, respectively. The age-standardised suicide rate increased by 42% between 2000 and 2017 (2000: 4.6, 95% CI 4.5–4.7; 2017: 6.5, 95% CI 6.4–6.6), whilst the age-standardised rate of combined suicide and undetermined intent decreased by 10% (2000: 11.8, 95% CI 11.6–11.9; 2017: 10.6, 95% CI 10.5–10.7).

**Fig. 1:**
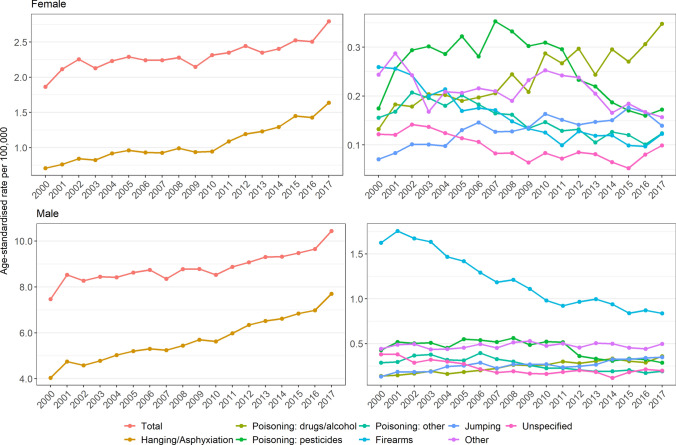
Age-standardised suicide rates per 100,000 by sex and method, 2000–2017

The total age-standardised suicide rate increased between 2000 and 2017 in both males and females (Fig. 1). In females, the total suicide rate increased by 50%, from 1.9 (95% CI 1.8–2.0) to 2.8 (95% CI 2.7–2.9) per 100,000. In males, the total suicide rate increased by 39%, from 7.5 (95% CI 7.3–7.7) to 10.4 (95% CI 10.2–10.7) per 100,000 in the same period. Total rates of death from combined suicide and undetermined intent increased by 4% in females, from 4.0 (95% CI 3.8–4.1) to 4.1 (95% CI 4.0–4.2) per 100,000, but decreased by 13% in males, from 20.0 (95% CI 20.0–20.3) to 17.4 (95% CI 17.1–17.7) per 100,000 (Figure S1). Rates of combined suicide and undetermined intent showed approximately similar trends to rates of suicide alone across all methods (Figure S2), although there appeared to be a slightly steeper decline in rates of firearms, other and unspecified methods between 2000 and 2017 for combined suicide and undetermined intent.

### Method-specific suicide rates

Of the total number of suicides during the period from 2000 to 2017 (*n* = 167,448), the most frequent method was hanging, accounting for 46.9% of all suicides in females and 64.8% in males (Table 1). Self-poisoning by each of drugs/alcohol, pesticides or other poisons accounted for a higher proportion of suicides in females (26.7%) than males (10.7%), whilst suicide by firearm was more frequent in males (13.4%) than females (6.3%).

**Table 1:** Incidence rates per 100,000, frequencies and percentages of suicides by method in Brazil in 2000, 2017, and change from 2000 to 2017

	2000–2017		2000		2017		Change: 2000–2017	
	*n* (%)	Rate (95% CI)	*n* (%)	Rate (95% CI)	*n* (%)	Rate (95% CI)	*n* (%)^a^	Rate difference
Total								
Any method	167,448 (100.0)	5.56 (5.54–5.58)	6658 (100.0)	4.59 (4.48–4.71)	12,450 (100.0)	6.53 (6.41–6.66)	5792 (87.0)	1.94
Self-poisoning: drugs/alcohol	7131 (4.3)	0.25 (0.24–0.25)	187 (2.8)	0.14 (0.12–0.16)	649 (5.2)	0.35 (0.33–0.38)	462 (247.1)	0.22
Self-poisoning: pesticides	10,334 (6.2)	0.35 (0.34–0.35)	406 (6.1)	0.30 (0.27–0.33)	445 (3.6)	0.23 (0.21–0.25)	39 (9.6)	− 0.07
Self-poisoning: other poisons	6161 (3.7)	0.21 (0.2–0.21)	311 (4.7)	0.22 (0.19–0.25)	310 (2.5)	0.16 (0.14–0.18)	− 1 (− 0.3)	− 0.06
Hanging/asphyxiation	102,196 (61.0)	3.39 (3.37–3.4)	3433 (51.6)	2.33 (2.25–2.42)	8660 (69.6)	4.59 (4.49–4.69)	5227 (152.3)	2.26
Firearms	19,976 (11.9)	0.66 (0.66–0.67)	1311 (19.7)	0.93 (0.87–0.98)	959 (7.7)	0.47 (0.44–0.50)	− 352 (− 26.8)	− 0.46
Jumping from high place	5947 (3.6)	0.20 (0.19–0.2.0)	147 (2.2)	0.10 (0.09–0.12)	463 (3.7)	0.24 (0.22–0.27)	316 (215.0)	0.14
Other	10,828 (6.5)	0.35 (0.34–0.35)	510 (7.7)	0.34 (0.31–0.37)	667 (5.4)	0.32 (0.30–0.35)	157 (30.8)	− 0.02
Unspecified	4875 (2.9)	0.16 (0.16–0.16)	353 (5.3)	0.25 (0.22–0.28)	297 (2.4)	0.15 (0.13–0.17)	− 56 (− 15.9)	− 0.10
Female								
Any method	35,365 (100.0)	2.33 (2.3–2.35)	1358 (100.0)	1.87 (1.76–1.97)	2656 (100.0)	2.8 (2.68–2.91)	1298 (95.6)	0.93
Self-poisoning: drugs/alcohol	3577 (10.1)	0.24 (0.24–0.25)	92 (6.8)	0.13 (0.11–0.16)	334 (12.6)	0.35 (0.31–0.39)	242 (263.0)	0.22
Self-poisoning: pesticides	3634 (10.3)	0.26 (0.25–0.27)	114 (8.4)	0.18 (0.14–0.21)	156 (5.9)	0.17 (0.14–0.20)	42 (36.8)	0.00
Self-poisoning: other poisons	2218 (6.3)	0.15 (0.15–0.16)	110 (8.1)	0.16 (0.13–0.19)	122 (4.6)	0.12 (0.10–0.15)	12 (10.9)	− 0.03
Hanging/asphyxiation	16,582 (46.9)	1.08 (1.06–1.09)	541 (39.8)	0.71 (0.65–0.77)	1518 (57.2)	1.63 (1.55–1.72)	977 (180.6)	0.93
Firearms	2214 (6.3)	0.16 (0.15–0.16)	174 (12.8)	0.26 (0.22–0.30)	111 (4.2)	0.12 (0.10–0.15)	− 63 (− 36.2)	− 0.14
Jumping from high place	2143 (6.1)	0.13 (0.13–0.14)	55 (4.1)	0.07 (0.05–0.09)	138 (5.2)	0.14 (0.12–0.17)	83 (150.9)	0.07
Other	3528 (10.0)	0.21 (0.21–0.22)	185 (13.6)	0.24 (0.21–0.28)	175 (6.6)	0.16 (0.13–0.18)	− 10 (− 5.4)	− 0.09
Unspecified	1469 (4.2)	0.09 (0.09–0.10)	87 (6.4)	0.12 (0.10–0.15)	102 (3.8)	0.1 (0.08–0.12)	15 (17.2)	− 0.02
Male								
Any method	132,083 (100.0)	8.97 (8.92–9.03)	5300 (100.0)	7.48 (7.28–7.69)	9794 (100.0)	10.43 (10.21–10.65)	4494 (84.8)	2.94
Self-poisoning: drugs/alcohol	3554 (2.7)	0.25 (0.24–0.26)	95 (1.8)	0.14 (0.11–0.17)	315 (3.2)	0.36 (0.32–0.40)	220 (231.6)	0.22
Self-poisoning: pesticides	6700 (5.1)	0.45 (0.44–0.46)	292 (5.5)	0.43 (0.38–0.48)	289 (3.0)	0.29 (0.25–0.33)	− 3 (− 1.0)	− 0.14
Self-poisoning: other poisons	3943 (3.0)	0.27 (0.26–0.28)	201 (3.8)	0.29 (0.25–0.33)	188 (1.9)	0.19 (0.17–0.23)	− 13 (− 6.5)	− 0.09
Hanging/asphyxiation	85,614 (64.8)	5.83 (5.79–5.87)	2892 (54.6)	4.05 (3.89–4.2)	7142 (72.9)	7.7 (7.51–7.89)	4250 (147.0)	3.65
Firearms	17,762 (13.4)	1.20 (1.18–1.21)	1137 (21.5)	1.62 (1.53–1.73)	848 (8.7)	0.84 (0.78–0.90)	− 289 (− 25.4)	− 0.79
Jumping from high place	3804 (2.9)	0.26 (0.25–0.27)	92 (1.7)	0.13 (0.11–0.16)	325 (3.3)	0.35 (0.31–0.39)	233 (253.3)	0.22
Other	7300 (5.5)	0.48 (0.47–0.50)	325 (6.1)	0.44 (0.39–0.50)	492 (5.0)	0.50 (0.45–0.55)	167 (51.4)	0.06
Unspecified	3406 (2.6)	0.23 (0.22–0.24)	266 (5.0)	0.38 (0.33–0.43)	195 (2.0)	0.20 (0.17–0.23)	− 71 (− 26.7)	− 0.18

*Rate* rate per 100,000, *CI* confidence interval

^a^Percentage values reflect the difference in number of suicides within a given stratum expressed as a proportion of suicides in 2000 for the stratum

From 2000 to 2017, the proportion of suicides by self-poisoning with drugs/alcohol showed the largest percentage increase (+ 247%, *n* = 462), followed by jumping from a high place (+ 215%, *n* =  + 316). The number of suicides due to hanging/asphyxiation increased substantially, with over 5200 more suicides from hanging in 2017 than 2000, reflecting a 152% rise in the proportion of all suicide deaths attributed to hanging. Firearm-specific suicides decreased over the period by approximately 27% (*n* =  − 352) and suicide by unspecified methods also decreased by 16% (*n* =  − 56).

Trends in rates of suicide by hanging/asphyxiation were roughly parallel to total suicide rates in both females and males. There were clear differences between males and females in patterns of suicide by drugs, pesticides and other poisons, firearms, and other methods (Fig. 1). Despite a considerable decrease in firearm-specific suicide in males, it remained the second most frequent method in 2017 (after hanging). Rates of suicide by self-poisoning from drugs/alcohol increased from 2000 to 2017, particularly in females. In females, the use of pesticides increased during the mid-2000s, and peaked in 2007, while in males, self-poisoning from pesticides was relatively stable between 2000 and 2011 and decreased after 2011.

Rates of method-specific suicide varied by age group (Figures S3 and S4). Between 2000 and 2017, males 60 years and older showed the largest increase in total suicide rates, followed by males 40–59 and 20–39, while females showed a relatively small increase in total suicide rates in all age groups. Hanging/asphyxiation showed the highest method-specific suicide rates in males and females of all age groups, which roughly paralleled total suicide rates. In females, the rate of self-poisoning by drugs and alcohol increased considerably among 20–59-year-olds. Rates of self-poisoning by pesticides were particularly high among females 10–39, although rates have decreased during the last decade. In males, self-poisoning by drugs and alcohol and jumping from a high place increased among people 20–39 years and older.

**Fig. 2:**
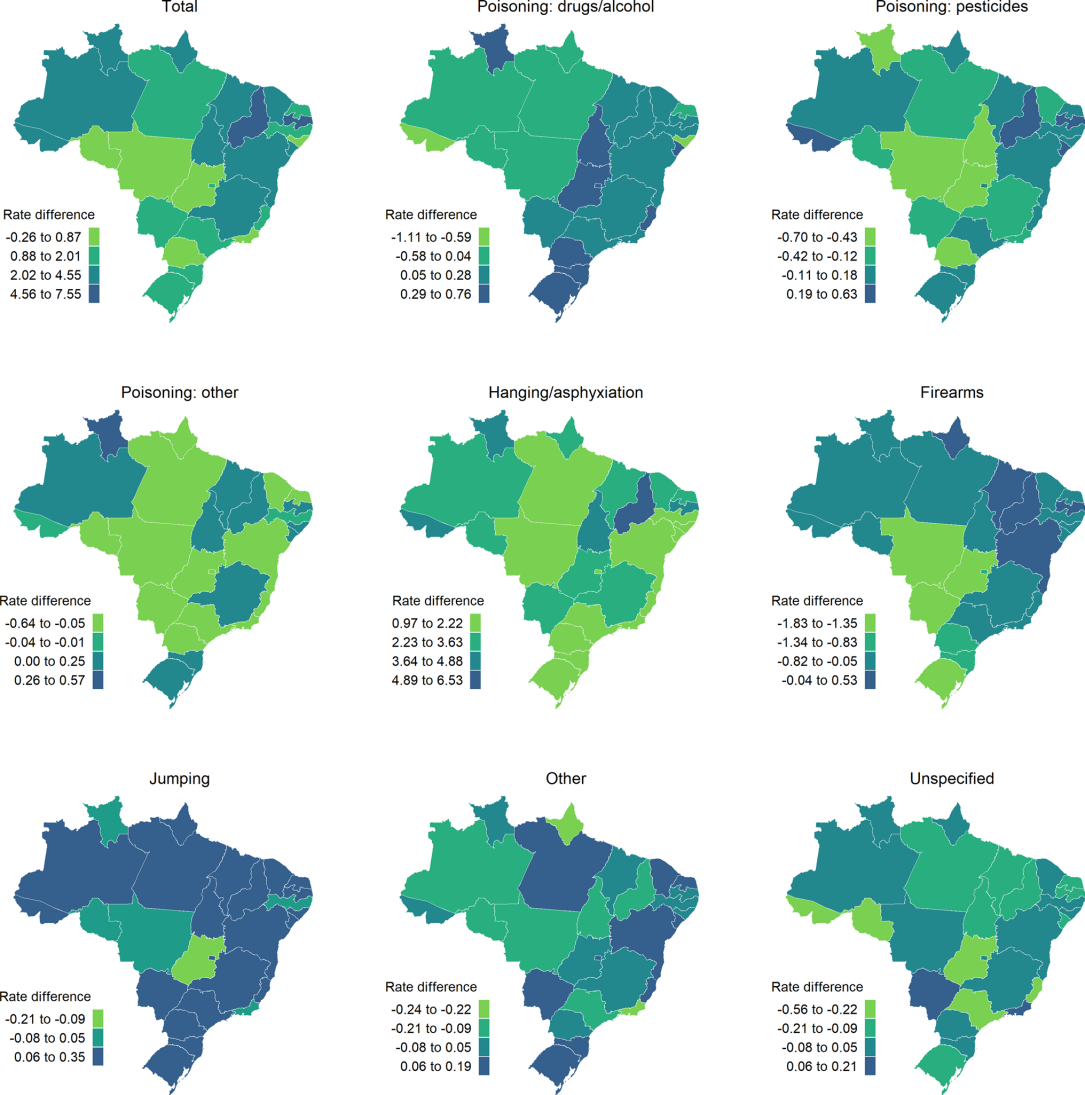
Change in age and sex-standardised rates per 100,000 of suicide by method, 2000 to 2017 for each Brazilian state. Data are absolute change in rate of suicide per 100,000 (rate difference)

We visualised the change in age and sex-standardised rates of suicide by method for each of the 27 states in Brazil from 2000 to 2017 (Fig. 2). The largest increases in total suicide rates were observed in Piauí (Rate difference [RD] = 7.5 per 100,000) and Paraíba (RD = 5.5 per 100,000). Piauí also showed the largest increase in rates of hanging/asphyxiation (RD = 6.5 per 100,000), along with several states in the North region. Firearm-specific suicide rates decreased in most states, particularly within the Central-West region. However, some states, including Piauí (RD = 0.5 per 100,000), Paraíba (RD = 0.4 per 100,000) and Maranhão (RD = 0.3 per 100,000) showed an increase in firearm-specific suicide rates. Visualisations of rates for each of 2000 and 2017 are shown in Figures S5 and S6, respectively.

### Firearm-specific suicide rates

**Fig. 3:**
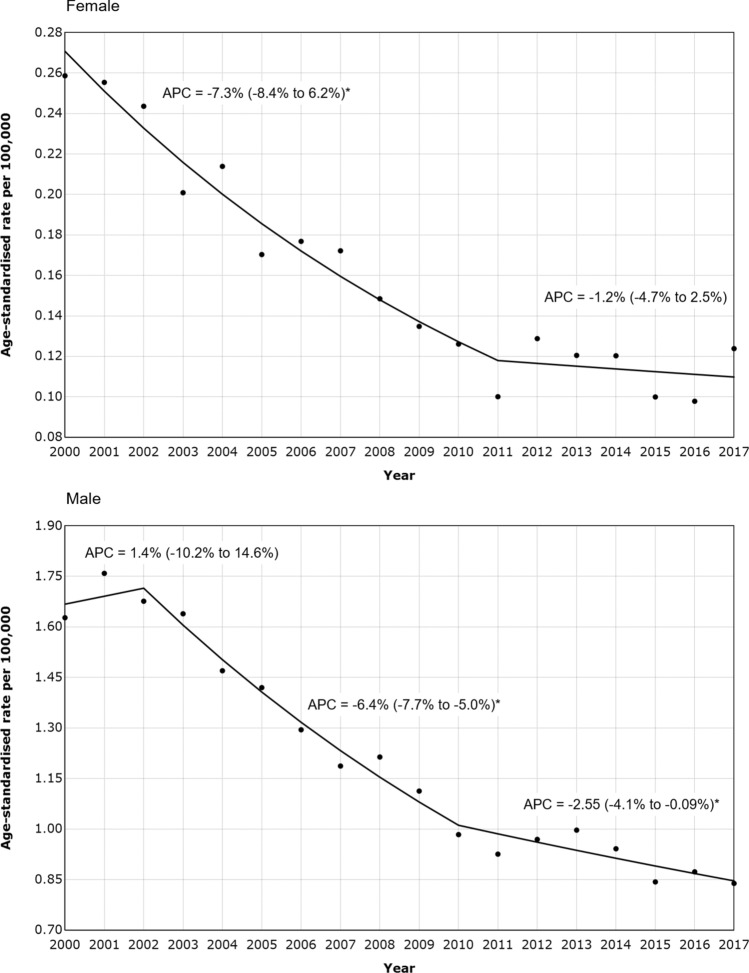
Trends in suicide by firearm by sex, 2000–2017. Trends in age-standardised rate of suicide by firearm per 100,000 in Brazil by sex, identified from the Joinpoint Regression analysis. *APC* annual percentage change. *Trend is significantly different from zero at the 0.05 level

The firearm-specific suicide rate generally decreased over the study period in both females and males (Fig. 3). From the Joinpoint Regression analysis, the firearm-specific suicide rate in females decreased annually by 7.3% per year between 2000 and 2011, and no clear change each year thereafter. In males, there was no significant change in firearm-specific suicide rate between 2000 and 2002, followed by a significant annual decrease of 6.4% from 2002–2010, and 2.5% from 2010 to 2017.

## Discussion

### Summary of main findings

Total age-standardised suicide rates have increased from 2000 to 2017. This finding is in line with the general increase of self-harm notifications in the country between 2009 and 2016 [19]. The total age-standardised rate of suicide in Brazil seems to be low compared to the average global suicide rate. For example, the age-standardised suicide rate for 2016 was 6.0 per 100,000 in Brazil, while the average global suicide rate in the same year was estimated as 11.2 per 100,000 [20]. However, when suicides verdicts are combined with deaths of undetermined intent, the rate (10.3 per 100,000) is considerably closer to the global average. Deaths from undetermined intent are widely recognised as “hidden” source of suicides [10, 21], therefore, the suicide rate in Brazil is likely to be higher than often reported based on suicide verdicts alone. We observed that the rate of suicide increased from 2000 to 2017, while the rate of undetermined intent decreased (as evidenced by the concurrent decreased in combined suicide and undetermined intent). Notably, deaths classified as undetermined intent likely contain a mixture of manners of death including homicide, suicide, and accidents, where the intent or origin of the injuries remains unclear. Therefore, the decreasing trend in deaths from undetermined intent could reflect a decrease in total deaths from external causes or improved classification of intent or both.

Rates of hanging/asphyxiation, self-poisoning by drugs/alcohol and jumping from a high place have shown the largest increases. Rates of firearm-specific suicides and unspecified methods decreased during the period. However, it is notable that changing trends may reflect changes in the absolute numbers of suicides as well as improvements in completion of death registrations. For example, it is unclear whether the reduced suicide rates from unspecified methods is due to better classification of injuries or a true shift away from the use of methods that fall within this category. The increase in total suicide rate suggests that this may reflect a shift away from firearms and unspecified methods and toward others, like hanging/asphyxiation and drugs/alcohol.

In line with the wider literature, we observed differences in the most commonly used methods by males and females. Whilst males showed consistently higher rates of overall suicide, there was a greater percentage increase in the suicide rate among females between 2000 and 2017. Females showed a higher proportion of suicides from self-poisoning and “other” methods, whilst firearm and hanging/asphyxiation contributed a much higher proportion of suicide deaths in males. Lethality may be higher in males due to comparatively greater levels of aggression and risk-taking, engagement in seemingly ‘sudden’ acts of self-harm, and selection of more lethal methods compared with females [22].

The examination of method-specific rates at state-level provides information about regional differences in methods of suicide. Changes in trends of different methods of suicide varied across states. For example, Paraíba and Piauí, showed particularly large increases in rates of hanging/asphyxiation and were also among the few areas to show increased firearm-specific suicide rates. Regional inequities in health care as well as differences in social and demographic characteristics may shape differential patterns. Paraíba and Piauí are amongst the poorest of Brazil’s 27 states, possibly contributing to the rising suicide rates in these areas. Future research should seek to examine smaller geographical regions and other factors that may influence choice of methods in Brazil. For example, it has been found that areas in Alagoas with higher agriculture and tobacco production show higher rates of self-poisoning from pesticides [23]. This may help to inform targeted public health interventions for populations at particularly high risk of certain methods of suicide.

Despite an increase in total suicide rates during the study period, firearm-specific suicides in Brazil decreased considerably between 2000 and 2017. The disarmament law was implemented in December 2003, which appears to be consistent with the pattern of firearm-specific suicide rates observed in this study; prior to 2002, males showed no evidence of a change in the rate of suicide by firearm; however, after this time, the rate decreased dramatically. Rates of suicide by firearm in females fell over the duration of the study, with no identified change in trend by the Joinpoint regression. Joinpoint regression assumes that the data follow a piecewise linear function, with segments connected continuously at unknown change points [15]. We chose this method given that the objectives were principally descriptive. Other methods that are available for the analysis of longitudinal trends include segmented or polynomial regression models. Segmented regression, which requires a priori specification of a hypothesised change point, may have also been appropriate given our interest in the implementation of the 2003 Disarmament Statute, however, we were also interested in other possible changes so preferred Joinpoint regression. Polynomial regression could be used if linearity assumptions were violated, but the data appeared to adequately fit the piecewise linear function. Finally, error may increase as the joinpoints approach the ends of the observation period [15]. Given that our analysis included relatively few observations prior to 2003, future studies with additional observations prior to 2000 may provide more accurate estimates.

Given the observed increase in total suicide rates coupled with decreased suicides by firearms, the displacement of method may have occurred such that limiting the availability of firearms led to the use of other methods, such as hanging. Notably, evidence suggests that a sharp decrease in total and firearm-specific homicide rates also occurred immediately following 2003; however, while suicide by firearms continued to decline through 2010, homicides by firearm increased again after 2005 [24]. Increased restrictions to firearms are a likely explanation for the observed decline after 2003; however, with continued enforcement, we would expect a continued decline in homicide rates as well.

Importantly, there were considerable differences in trends of firearm-specific suicide rates across states; whilst some areas observed decreasing trends, others showed increased suicides by firearm over the study period. These differences can potentially be explained by local variation in the implementation and enforcement of the 2003 national gun-control laws (and, therefore, differences in the availability of firearms), among other factors [25], which may also have affected the distribution of suicides by firearm across Brazilian states. Indicators of the availability of firearms show important differences in trends between Brazilian states from 2000 to 2010, with reduced prevalence of firearms in the states where homicides have also declined versus increased prevalence in states where homicides went up [24]. It appears that the trends in suicide rates observed in our study are roughly consistent with changes in prevalence of firearms and homicide rates at the state-level. For example, Pernambuco, São Paulo, and Rio de Janeiro were reported to have the largest reductions in prevalence of firearms from 2000 to 2010 and also showed decreased firearm-specific suicide rates within our studies. Likewise, Pará, Maranhão, and Bahia had the largest increases in firearm prevalence and were also among the few states that showed increased firearm-specific suicide rates [25]. In line with these findings, a study using data from the Global Burden of Disease study showed that rates of firearm-related deaths are associated with the numbers of firearms voluntarily returned following implementation of the Disarmament Statute; while rates of firearm-related deaths tended to increase between 2000 and 2017 in states with low numbers returned, states with the highest numbers of firearms returned showed reductions in the rates of firearm-related deaths [6].

### Strengths and limitations

This study examined data from a nationally representative database that contains information from the death certificate, which enabled a detailed examination of trends in methods of suicide in Brazil. Nevertheless, our study has some limitations.

One limitation of this study is potential misclassification of suicide deaths. Suicides are widely believed to be underreported and may be misclassified as deaths of other causes such as undetermined intent or accidents, particularly when suicidal intent cannot be determined. Furthermore, religious and cultural beliefs against suicides, concerns of stigma, political pressure and insufficient physical and psychological autopsy procedures can lead to alternative verdicts. Whilst seen as less frequent, it is also possible that some deaths from other causes, like homicides, may also be misclassified as suicides under some circumstances (for example, in young marginalised women [26]). Changes in completeness of data and accuracy of reporting over time may have contributed to the temporal patterns observed. To investigate one potential source of misclassification, we also examined deaths due to undetermined intent. Whilst an increase in total suicide rates occurred alongside a decreasing trend in rates of undetermined intent, misclassification does not appear to fully explain the rise in total suicide rates in Brazil. An international assessment of quality of cause-of-death data conducted in 2003 classified Brazil’s quality of death registration as intermediate [27] and more recent analyses of the Brazilian SIM have shown that the quality of these data have been recognised for having high-quality standards [28, 29]. Quality and completeness of death registrations has also been found to vary considerably across Brazil [30], which may have influenced the state-level analysis, and therefore, should be interpreted with some caution.

Additionally, the population estimates used in this study were linear projections based on census population estimates, which may not accurately capture the annual variations in population, and in turn, could influence the estimated mortality rates. This was necessary because population estimates stratified by age, sex, and state were only available for a subset of the years in this study. However, we have examined a relatively rare outcome in relation to a very large population denominator, therefore, the estimated rates will be quite robust to variations in the populations.

### Implications

This work documents the temporal trends and geographical distribution of methods of suicide in Brazil, which is useful for health and social service providers and policy makers. The apparent rise in suicide rates in Brazil highlights the need for effective public and social health strategies. Improved understanding of trends in methods of suicide can inform targeted suicide prevention. Reductions of suicides following detoxification of domestic gas supplies [31] and restrictions to pesticides in Sri Lanka [32] are examples of effective public health strategies aimed at reducing access to commonly used suicide methods, and highlight the potential positive impact of means restriction. Surveillance of methods of suicide may also help to identify a rise in new common methods of suicides, which could become targets for prevention.

Although limiting access to means can be an effective strategy, this is only one method of prevention. Importantly, access to certain means of suicide, such as hanging, is not easy to limit. Further, substitution of means (i.e. switching to other methods of suicide) after restriction may be of concern. In this study, the fall in the rate of firearm-specific suicide coincided with an increase in hanging/asphyxiation. Restriction of means is most effective if the method is highly available and lethal, and not easily substituted for another method, and when implemented in conjunction with other suicide prevention strategies [33]. Suicide prevention should focus on the prevention of psychiatric disorders, and on the social and environmental risk factors for suicide [34]. Given the rise in the use of self-poisoning by drugs or alcohol in Brazil, suicide prevention policies in the country should also include strategies on assessment and management of alcohol and substance use. National studies in Brazil have shown strong associations between suicidal ideation and attempts, and alcohol use disorder (as defined by the Diagnostic and Statistical Manual, 5th Edition [35]), marijuana and cocaine use [36]. Therefore, improved health and social programmes aimed at prevention and management of alcohol and drug misuse, may also help to reduce suicide by these methods.

Given the high prevalence of gun violence in Brazil, restricting access to firearms is one particularly salient strategy for reducing deaths. Rates of firearm-specific suicide decreased over the last 2 decades, and the timing of changes in trends coincided with the implementation of the Disarmament Statute. Our findings are consistent with other evidence supporting the association between stricter gun-control and the reduction of firearm-related deaths, including suicides, in Brazil and other countries [7, 8, 37, 38], with particularly successful results in Australia [39] and New Zealand [40] following regulatory reforms. If this is a causal association, then greater control over access to firearms will contribute a positive impact on suicide prevention. Benefits may also extend to other areas of public health and the prevention of violence. It will be important for future research to examine changes in firearm-related suicides given an amendment to the Disarmament Statute in 2019, which facilitates access to and possession of firearms in Brazil (Decree No. 9685 of January 15, 2019) [41]. It can be hypothesised that this new legislation may slow or reverse the decreasing trend in firearm-related suicide and will require future investigation. Future research tracking changes in the relaxation of firearm access could improve our understanding of the relationship between firearm regulation and suicide in Brazil.

## Supplementary Information

Below is the link to the electronic supplementary material.

Supplementary file1 (PDF 5427 KB)

## Data Availability

All data used in this study are publicly available online (http://www2.datasus.gov.br).

## Code availability

Code is available by request to the corresponding author.
