# Supplementary material for: Trends in method-specific suicide in Brazil from 2000 to 2017
Source: Soc Psychiatry Psychiatr Epidemiol. 2021 Mar 29;56(10):1779–90. doi: 10.1007/s00127-021-02060-6 (PMC8429168; doi:10.1007/s00127-021-02060-6)

Supplemental Material

Trends in method-specific suicide in Brazil from 2000 to 2017

Keltie McDonald[1], Daiane Borges Machado[2,3], Luís F S Castro-de-Araujo[2,4], Lígia Kiss[5], Alexis Palfreyman[5], Maurício L. Barreto[2,6], Delanjathan Devakumar[5], Glyn Lewis[1]

[1] Division of Psychiatry, University College London, London, United Kingdom.
[2] Center of Data and Knowledge Integration for Health (CIDACS), Salvador, Brazil.
[3] Department of Global Health and Social Medicine, Harvard Medical School, Boston, Massachusetts, USA.
[4] The University of Melbourne, Department of Psychiatry, Austin Health, Heidelberg, Victoria, Australia.
[5] Institute for Global Health, University College London, London, United Kingdom.
[6] Institute of Collective Health, Federal University of Bahia, Salvador, Brazil.

Contents

Section 1: Missing data for suicide cases: Additional details.
Table S1. Missing data on age, stratified by method and sex.
Figure S1. Age-standardised rates of death from suicide and undetermined intent per 100,000 for males and females, 2000-2017.
Figure S2. Age-standardised rates of death from suicide and undetermined intent per 100,000 for males and females by method, 2000-2017.
Figure S3. Age-standardised suicide rates per 100,000 in females ages 10-19, 20-39, 40-59 and 60 years and older by method, 2000-2017.
Figure S4. Age-standardised suicide rates per 100,000 in males ages 10-19, 20-39, 40-59 and 60 years and older by method, 2000-2017.
Figure S5. Age and sex-standardised suicide rates per 100,000 by state, 2000.
Figure S6. Age and sex-standardised suicide rates per 100,000 by state, 2017.

Section 1: Missing data for suicide cases: Additional details

Missing data for deaths classified as suicide were explored in more detail. There were few records missing sex information, however missing age information may have influenced results, therefore summarised the frequencies and proportions of cases missing age information, by sex and method (Table S1). Other and unspecified methods of suicide tended to have more cases with missing age. Males also had a slightly higher proportion of cases missing age than females. The number of cases missing age also tended to decrease over the study period (data not shown here), from approximately 1.7% to 0.3% across any method (data not shown here).

Table S1. Missing data on age, stratified by method and sex.

	Both sexes			Male			Female		
Any method	Total cases	Cases missing age	% missing age	Total cases	Cases missing age	% missing age	Total cases	Cases missing age	% missing age
Poisoning: Drugs/alcohol	168703	1246	0.74	133128	1045	0.78	35550	185	0.52
Poisoning: Pesticides	7164	33	0.46	3577	23	0.64	3587	10	0.28
Poisoning: Other poisons	10374	39	0.38	6728	28	0.42	3645	11	0.30
Hanging/asphyxiation	6220	59	0.95	3988	45	1.13	2232	14	0.63
Firearms	102906	704	0.68	86227	613	0.71	16661	79	0.47
Jumping from high place	20071	94	0.47	17850	88	0.49	2220	6	0.27
Other	6017	70	1.16	3862	58	1.50	2154	11	0.51
Unspecified	11000	172	1.56	7428	128	1.72	3570	42	1.18

[a] Total cases include a small number of cases with missing sex information, there male and female columns do not sum to 100%.

Figure S1. Age-standardised rates of death from suicide and undetermined intent per 100,000 for males and females, 2000-2017.

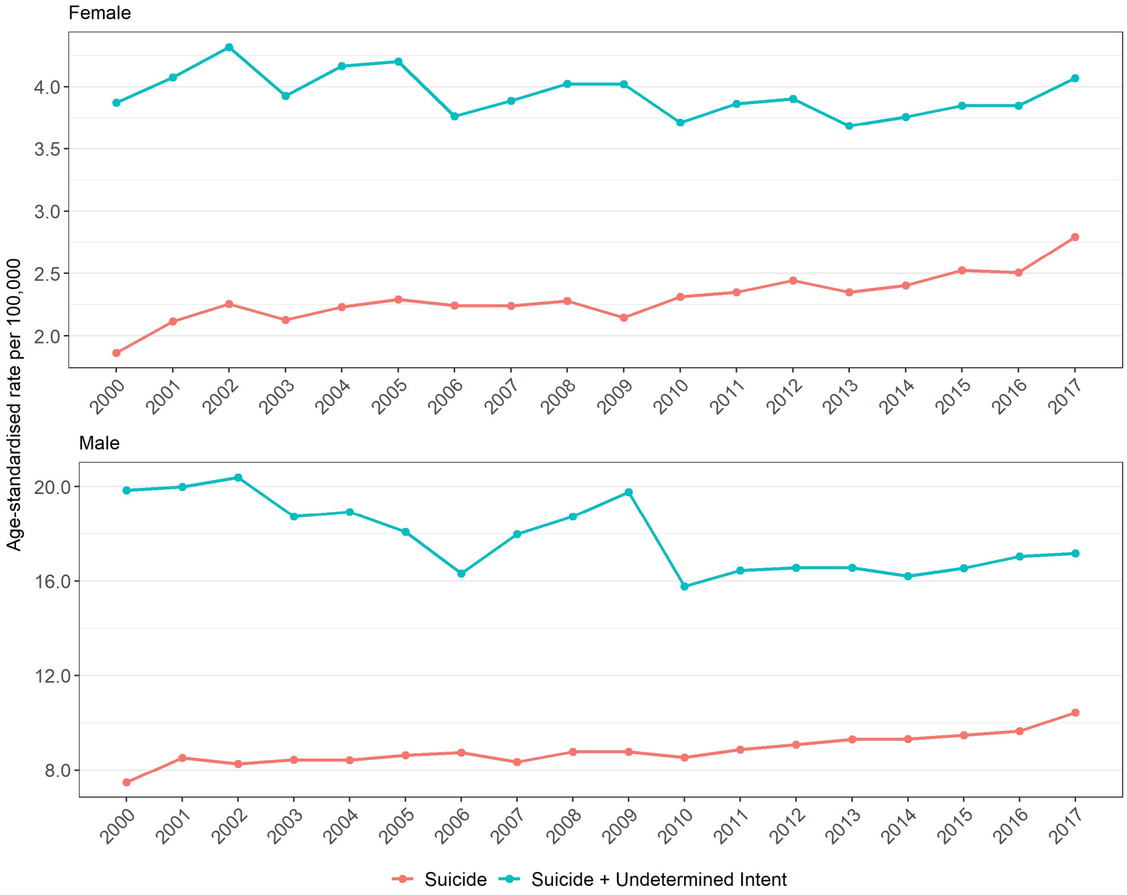

Figure S2. Age-standardised rates of death from suicide and undetermined intent per 100,000 for males and females by method, 2000-2017.

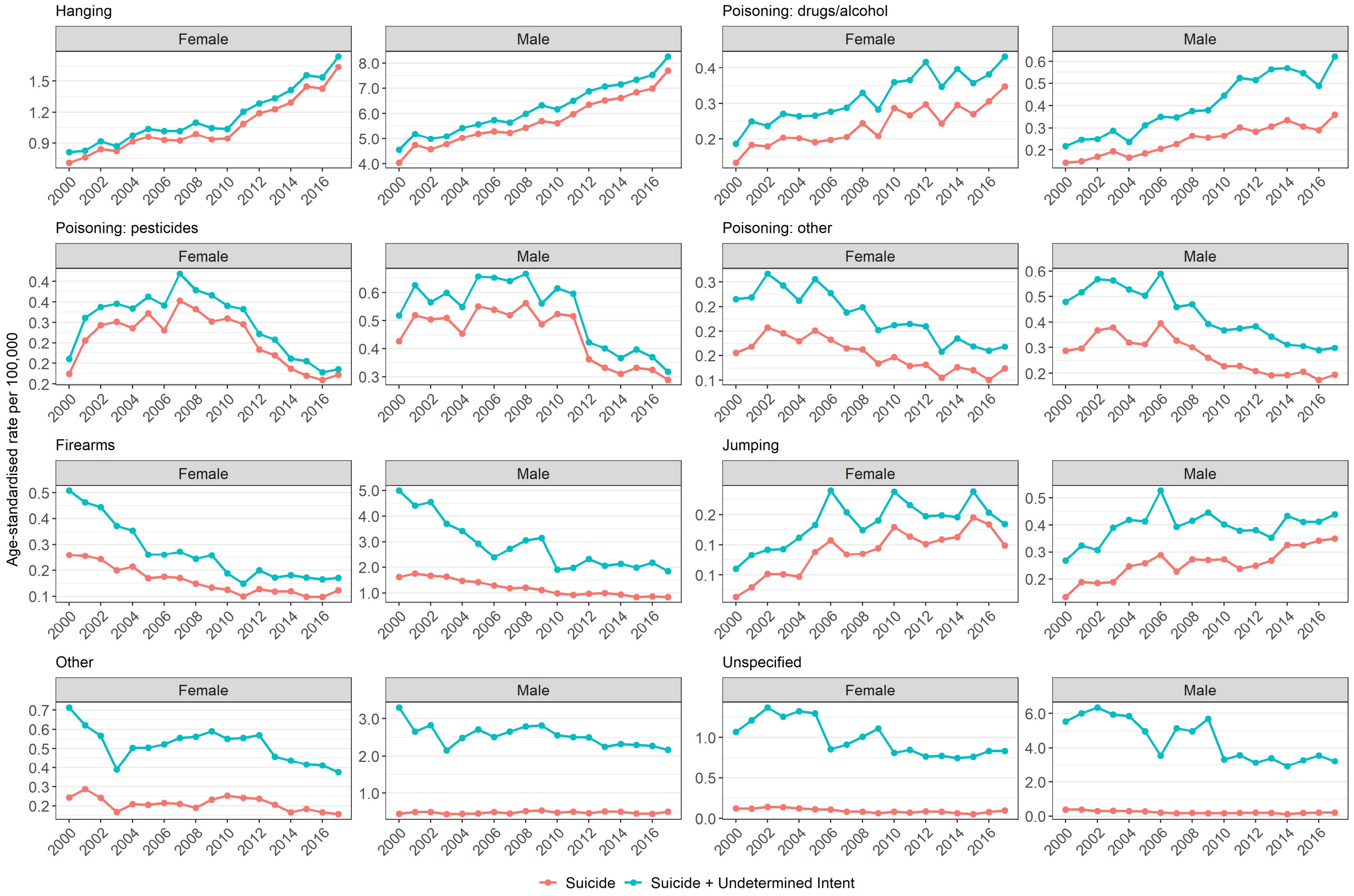

Figure S3. Age-standardised suicide rates per 100,000 in females ages 10-19, 20-39, 40-59 and 60 years and older by method, 2000-2017.

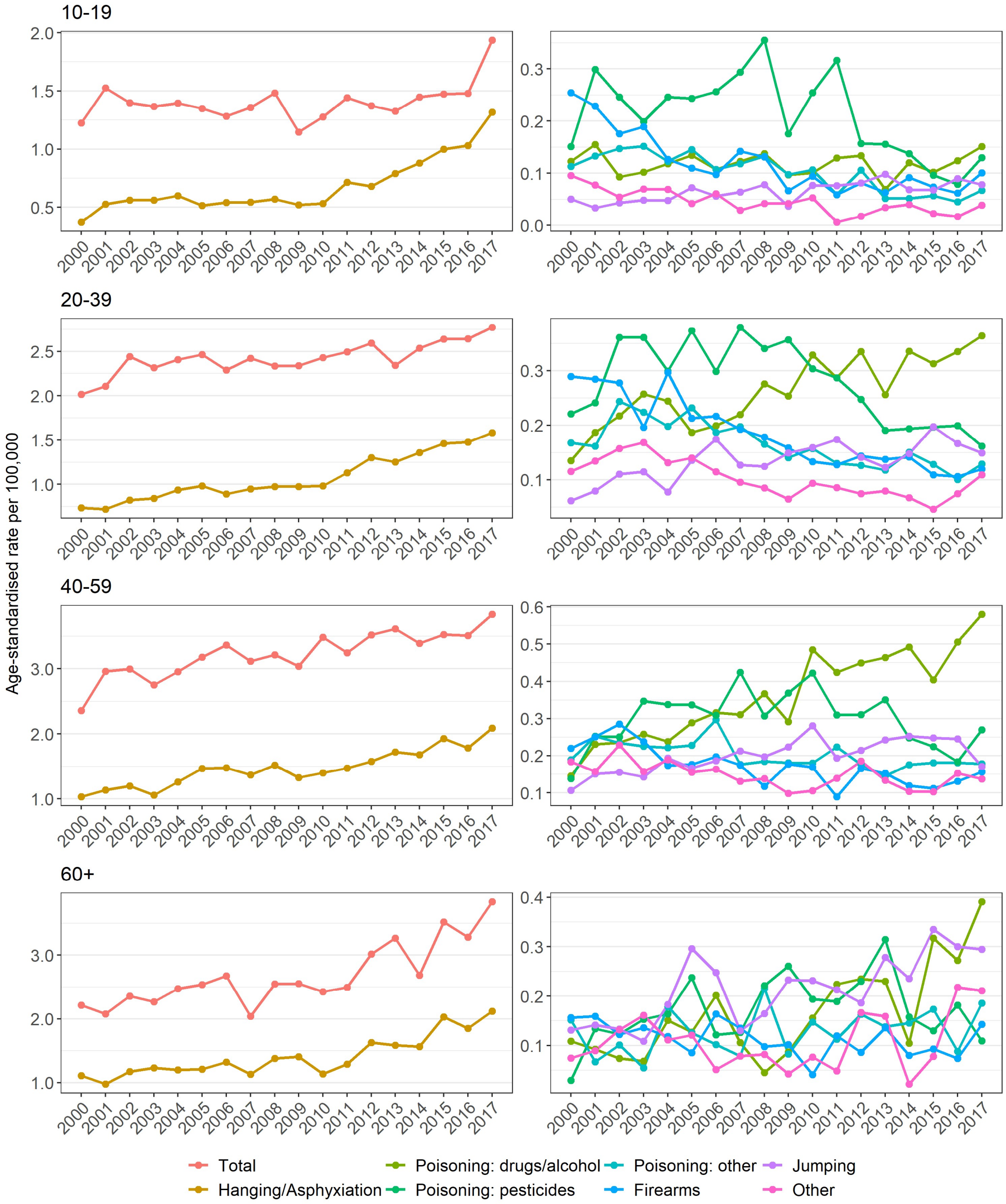

Legend: Rates are age-standardised within broad age groups.

Figure S4. Age-standardised suicide rates per 100,000 in males ages 10-19, 20-39, 40-59 and 60 years and older by method, 2000-2017.

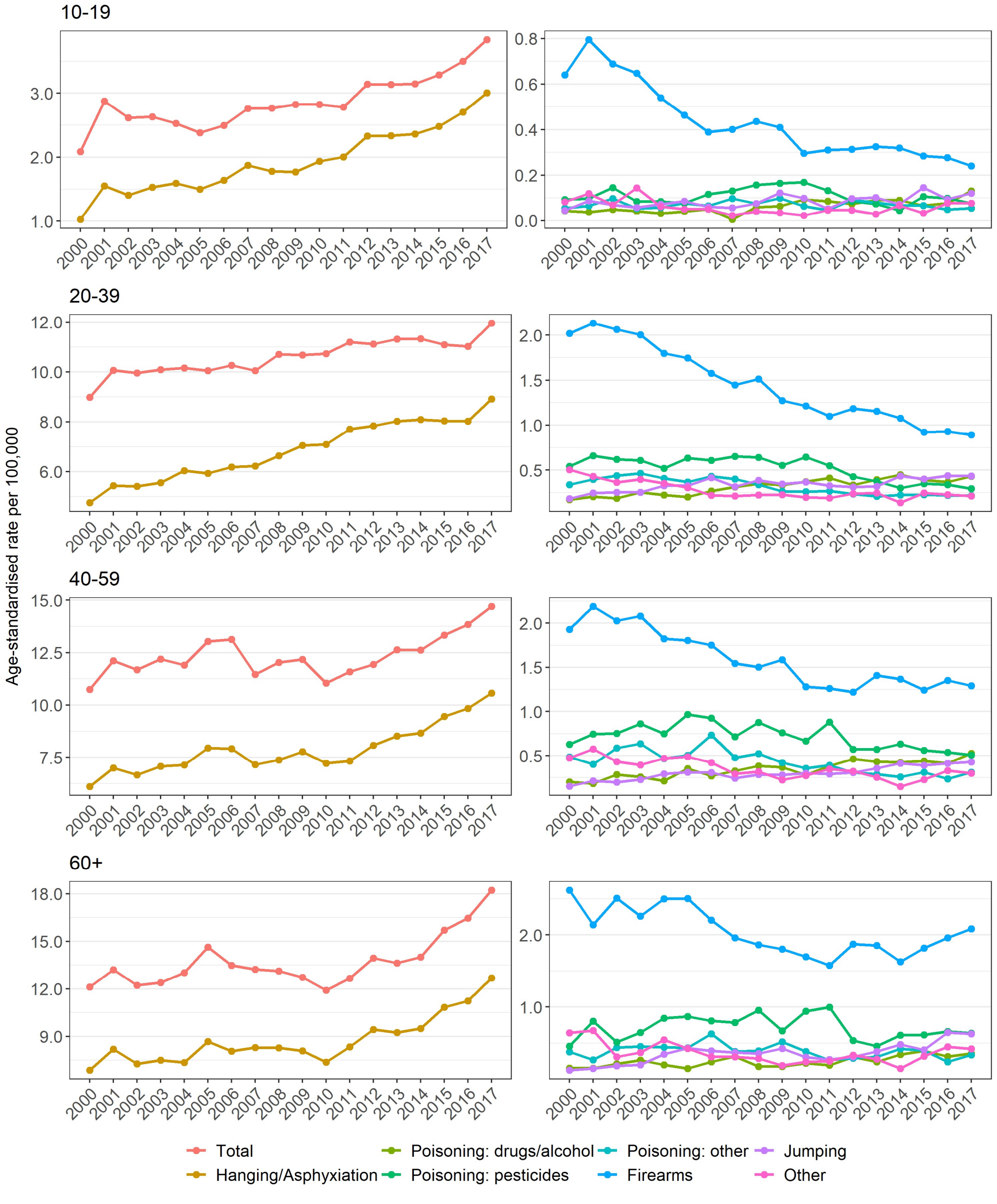

Legend: Rates are age-standardised within broad age groups.

Figure S5. Age and sex-standardised suicide rates per 100,000 by state, 2000.

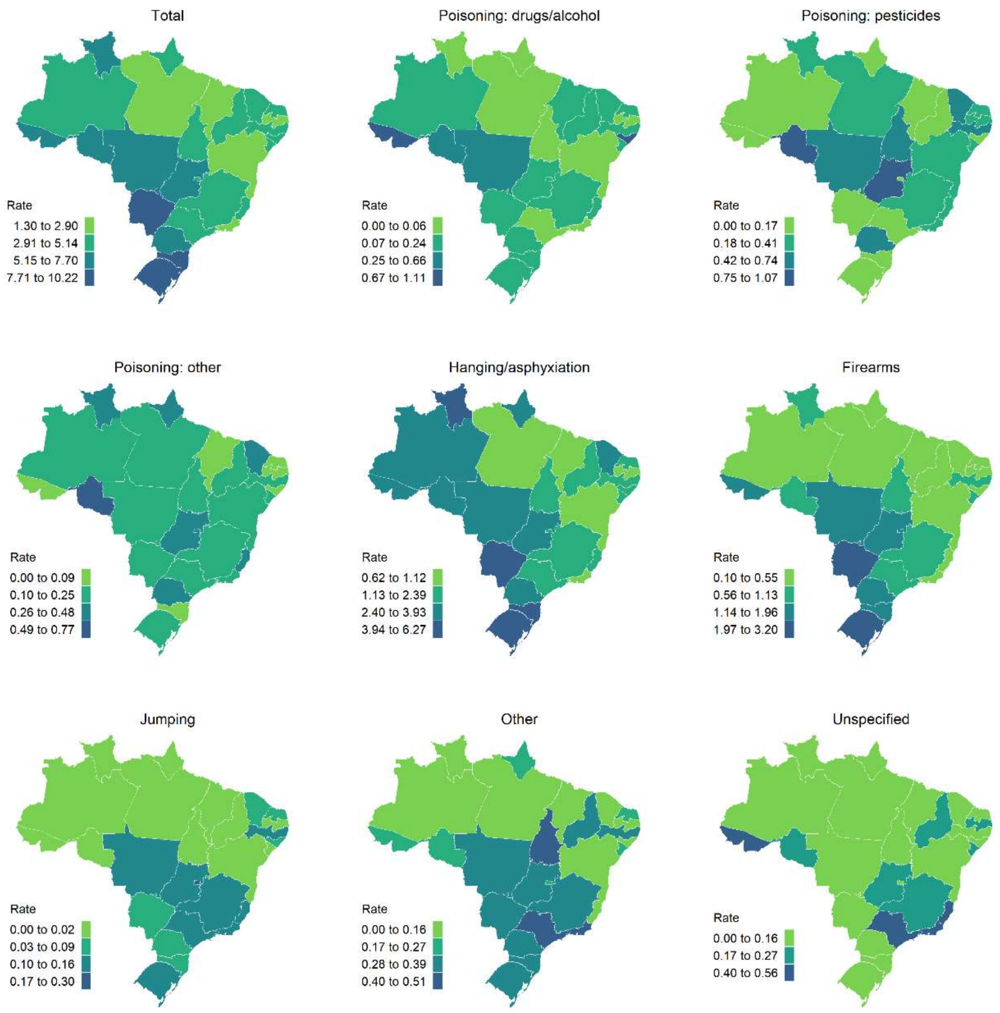

Figure S6. Age and sex-standardised suicide rates per 100,000 by state, 2017.

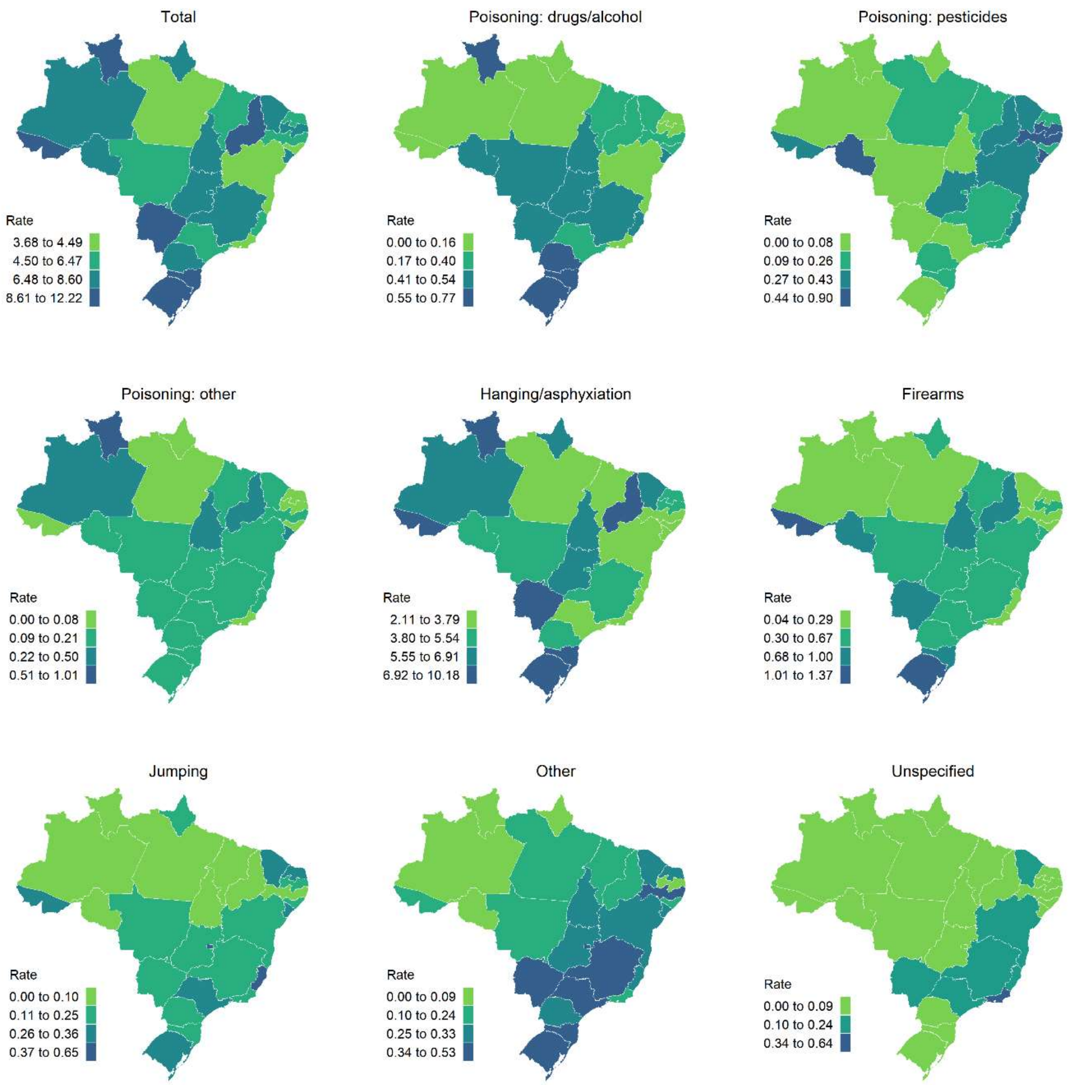

Supplement: Supplementary file 1 — Supplementary file1 (PDF 5427 KB) [file 127_2021_2060_MOESM1_ESM.pdf]
